# Determination of Some Heavy Metals in European and Polish Coal Samples

**DOI:** 10.3390/molecules28248055

**Published:** 2023-12-12

**Authors:** Bożena Karbowska, Ewelina Włódarzewska, Włodzimierz Zembrzuski, Joanna Zembrzuska, Edyta Janeba-Bartoszewicz, Jarosław Bartoszewicz, Jarosław Selech

**Affiliations:** 1 Faculty of Chemical Technology, Poznan University of Technology, Berdychowo 4 St., 60-965 Poznan, Poland; bozena.karbowska@put.poznan.pl (B.K.); ewelina.blaszkow@maxcess.eu (E.W.); wlodzimierz.zembrzuski@put.poznan.pl (W.Z.); joanna.zembrzuska@put.poznan.pl (J.Z.); 2 Faculty of Civil and Transport Engineering, Poznan University of Technology, Piotrowo 3 Str., 60-965 Poznan, Poland; jaroslaw.selech@put.poznan.pl; 3 Faculty of Environmental Engineering and Energy, Poznan University of Technology, Piotrowo 3 Str., 60-965 Poznan, Poland; jaroslaw.bartoszewicz@put.poznan.pl

**Keywords:** heavy metals, pollution, electrochemical measurements

## Abstract

This work presents coal analyses for heavy metal content (Tl, Cu, Zn, Cd, Fe). The tested coal samples came from a Russian deposit in the Kuzbass Basin (Novosibirsk and Kemerovo Oblasts, near Kazakhstan) and from Poland. The concentration of thallium in coal was determined using DPASV—differential pulse anodic stripping voltammetry—and other metals were examined with FAAS, i.e., flame atomic absorption spectrometry. The study confirmed the presence of thallium in the tested coal sample. The coal samples from outside the European Union contained four times more thallium (the maximum content of thallium in coal has been determined to be 0.636 mg·kg^−1^) than the samples of Polish coal (where the maximum content of thallium was 0.055 mg·kg^−1^). Cadmium concentration was on average 1.99 mg·kg^−1^ in the samples from outside the European Union, and 1.2 mg·kg^−1^ in the samples of Polish coal. Zinc concentration in the samples from outside the European Union was on average 11.27 mg·kg^−1^, and in the samples of Polish coal approx. 7 mg·kg^−1^. In addition, iron concentration in all coal samples was determined as 14.96 mg·kg^−1^, whereas copper concentration in the samples from outside the European Union averaged as 3.96 mg·kg^−1^. The obtained results do not show any correlation between the presence of thallium and the presence of other metals. It is worth noting that heavy metals pose a threat to living organisms due to their persistence and bioaccumulation, particularly in the context of dust emissions to the atmosphere.

## 1. Introduction

The problems with hard coal are concerning at the moment and require a detailed analysis of various aspects of its use. The European Union, with this year’s directive, decided to suspend the pace of coal withdrawal from the energy sectors of the member states. However, it must be related to the analysis of the chemical composition of coal, and in particular, the heavy metal content. Coal contains many heavy metals, as it is created through compressed organic matter containing virtually every element in the periodic table—mainly carbon, but also heavy metals. The chemical structure of hard coal is related to many chemical compounds found in coal (organic and inorganic). Taking into account the physicochemical structure of coal, it can be concluded that it is very complex and still being studied. Due to the different behavior of these compounds during combustion, it is possible to distinguish substances forming flammable substances and ballast. Ballast includes mineral compounds that form ash during combustion. The combustible substance of coal consists of hydrocarbons and other organic compounds consisting of oxygen, nitrogen, and sulfur. The combustion processes of hard coal (as well as other fuels) are the main causes of air pollution. The amount of waste generated depends mainly on the amount of coal burned and its quality, as well as on the types of furnaces used. Coal is currently controversial in light of greenhouse gas emissions, but its relatively low price still speaks in its favor. At the present time, the extraction and use of coal is in decline due to the relative emissions of organic and inorganic pollutants to the atmosphere. This applies particularly to the emission of heavy metals that pose a significant threat to plant, animal, and human organisms.

According to EUROCOAL (the European Coal and Lignite Association, based in Brussels), the known global resources are around 400 billion tonnes. According to the data provided by the Polish Geological Institute, as much as 80% of Polish coal resources are in Upper Silesia. All coal combustion products and those related to its extraction are considered polluting, with a distinction being made between volatile and solid by-products including, e.g., mercury in fuels [1]. Metals in coal are among the most dangerous pollutants in the environment due to their persistence, bi-accumulation, and acute toxicity. Cadmium, lead, and thallium are ingredients that are potentially hazardous to health and have been identified by the U.S. Environmental Protection Agency.

The mechanism of Tl toxicity involves a disruption of enzymatic activity and metabolic processes. Symptoms of Tl poisoning typically include hair loss, ulcers, internal bleeding, myocardial damage, alopecia, poly-neuropathy, insomnia, paralysis, weight loss and, ultimately, death [2]. Cd is neurotoxic, carcinogenic, and mutagenic. Tl is neurotoxic, carcinogenic, and mutagenic [3]. Zinc, copper, and iron, on the other hand, are essential for the proper course of key biological processes. Iron in the form of heme is part of numerous enzymes that protect cells against oxidative stress. Zinc is a component of DNA and RNA polymerases, and is also responsible for the processes of replication and transcription of genetic material and is involved in gene expression. Copper is found in all tissues in the body and plays an important role in the production of red blood cells and nerve cells and stimulates the immune system. It helps in the formation of collagen, iron absorption, and energy production [3].

This work presents a coal analysis for heavy metal content (Tl, Cu, Zn, Cd, Fe). The tested coal samples came from a Russian deposit in the Kuzbass Basin (Novosibirsk and Kemerovo Oblasts, near Kazakhstan) and the Polish ones from the deposits of the Polish Upper Silesian Basin. Thallium concentration was determined by the method of differential pulse anodic stripping voltammetry—DP-ASV. Due to its high sensitivity, relatively expensive equipment and a variety of uses, important electrodes, and the possibility of their modification, for a long time it has been considered a powerful technique for determining trace metals ducts (including thallium). Copper, zinc, cadmium, and iron determinations were carried out using the flame atomic absorption spectrometry (FAAS) technique. FAAS techniques have the advantages of high sensitivity, wide analysis range, a simple instrument, and the automation of the whole operation, as well as accurate and reliable results. Moreover, FAAS is characterized by a lower sensitivity to the influence of the matrix compared to methods for separation and spectral parameters and allows for conducting real-time analyses. These advantages make FAAS unrivalled in the field of heavy metal analysis and detection, but FAAS is an expensive instrument and has high operating costs, which may limit its application [2].

## 2. Experimental Research Conditions

### 2.1. Materials

Coal samples were sourced from the deposits of the Polish Upper Silesian Basin (9 ÷ 10, Table 1), and from the Novosibirsk district in Russia (1 ÷ 8, Table 1), obtained on the local market. All samples were ground and powdered with an agate mortar. The powdered samples were passed through a 60-mesh sieve. The sieved material was dried at 50 °C and stored in plastic bags. In the first stage of thallium determination, the samples were mineralized. Then, they (0.25 g) were placed in a Teflon beaker and digested by adding 4 mL of 73% HF, 65% nitric acid, 2 mL of HClO_4_, and 2.5 mL of 30% hydrogen peroxide. After the solution was evaporated, the residue was mixed with an additional dose of nitric acid (2 mL), covered with a glass slide, and heated for 2 h. After filtration, the residue was mixed with ascorbic acid (2.5 mL of a 1 M solution) and EDTA (6.25 mL of a 0.2 M solution). Then, the pH of the solution was adjusted to 4.5 (with ammonia solution), added to a flask (25 mL) and made up with distilled water. The obtained solutions of coal samples were used for thallium determination following the method of differential pulse anodic stripping voltammetry (DPASV). The limit of detection of the method (calculated based on 3SD) was 50 pg·L^−1^ (0.25 pM) [2,3,4].

### 2.2. Solutions and Preparations

Standard solutions of Tl and other analyzed metals were prepared by dilution of a 1000 μg·mL^−1^ stock standard solution obtained from Sigma Aldrich (Burlington, MA, USA). Ammonia solution (25%), nitric acid (65%), hydrofluoric acid (73%), hydrogen peroxide (30%), EDTA, and ascorbic acid (supplied by Sigma Aldrich) were used to conduct the determination. All solutions were prepared in high-purity water obtained by reverse osmosis in a Watek-Demiwa 5 Rosa system, followed by a triple distillation from a quartz apparatus. Only freshly distilled water was used. The following reference materials were used in this work: NCS DC 73382—Chinese National Standard Reference Materials, Beijing.

### 2.3. Instruments

**Figure 1:**
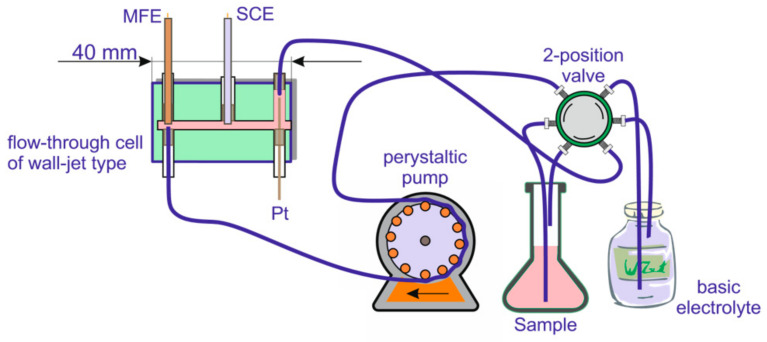
A three-electrode measuring cell, consisting of a mercury film working electrode (MFE) based on glassy carbon, a saturated calomel reference electrode (SCE), and a platinum wire auxiliary electrode.

A μAutolab electrochemical analyzer from EcoChemie (Utrecht, The Netherlands) was used for electrochemical measurements. The determination of thallium in coal samples was conducted using a previously described procedure [3,4]. Thallium concentration was determined using the method of pulse-differential anode stripping voltammetry—DP-ASV. Measurements were made in the deoxygenated environment with 0.05 M EDTA pH 4.5, which is the primary electrolyte. The tests were conducted under the following conditions: concentration time from 900 s; concentration potential: −0.9 V; differential pulse amplitude: 50 mV; step potential: 2 mV; starting potential: −0.9 V; end potential: −0.4 V; scan frequency: 0.01 V s; differential pulse time: 0.07 s; time to settle the solution: 10 s. The measuring system (see Figure 1) was equipped with a peristaltic pump with a flow rate of 20 mL/min, which delivered electrolytes to a flow bowl with three electrodes: working (mercury film electrode), reference (calomel electrode), and auxiliary (platinum electrode). Before starting the measurement, the electrode was subjected to mechanical cleaning with the use of aluminum oxide (Al_2_O_3_) suspension on a soft surface. In order to clean the electrode from the remains of adsorbed Al_2_O_3_, the electrode was placed in a vial filled with redistilled water, then transferred to an ultrasonic bath and left for a few minutes. The use of a flowmeter system eliminated the problem of thallium running out from the sample solution. The system was continuously deoxidized with a stream of purified nitrogen. EDTA (0.05 M) was used as a primary electrolyte. Lead and thallium exhibit similar electrochemical properties. As a result, overlapping voltammetry signals from both metals are usually observed in the systems that do not contain complexing electrolytes. The problem, however, may be easily avoided by adding EDTA. The EDTA complex with Pb^2+^ is very stable and hampers the reduction of Pb^2+^ to the metallic state, thus minimizing the interference of this ion. Due to the use of EDTA as a supporting electrolyte, the method tolerates a 1000-fold excess of lead [3,4]. The metal ion concentrations (Cd, Zn, Cu, Fe) were measured using flame-atomic absorption spectrometry (FAAS) with a Z-8200 spectrometer equipped with a premix fishtail-type burner air/acetylene and NO_2_/acetylene, graphite furnace-flame, and furnace on the same beam made by Hitachi, Japan.

## 3. Results and Discussion

**Figure 2:**
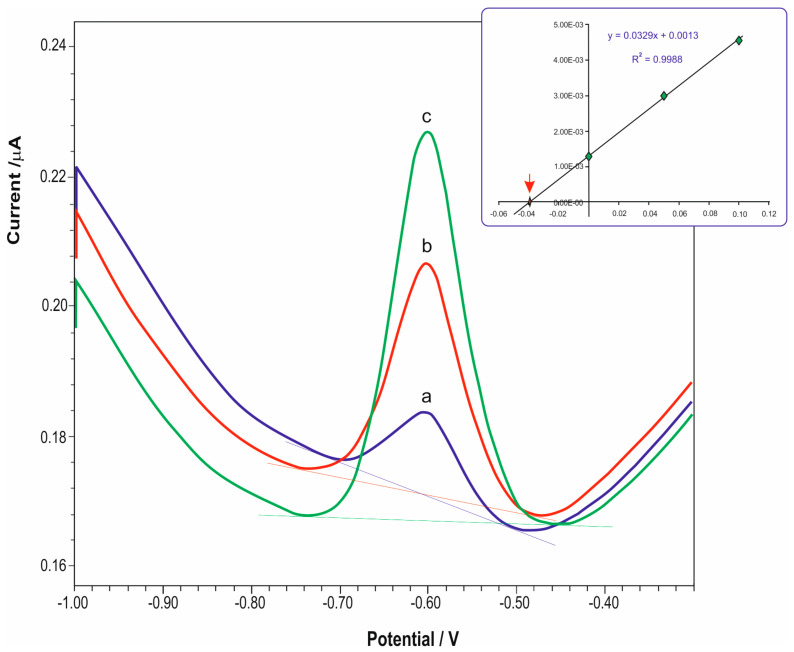
Voltametric curve for the coal sample and for (**a**) the coal sample with sequential standard addition, (**b**) 0.05 ppb Tl, and (**c**) 0.1 ppb Tl. The supporting electrolyte was 0.05 M EDTA (pH = 4.5). Pre-concentration potential of −0.9 V vs. Ag/AgCl, pre-concentration time of 900 s, pulse amplitude of 50 mV, step potential of 2 mV. Standard deviation of the measured value of Tl concentration.

Thallium concentration in the samples was determined on the basis of several standard additions. Figure 2 shows an exemplary voltamperogram for the sample and the sample with sequential standard addition at two concentration levels. Figure 2 shows an exemplary multiple standard addition curve. Each sample was measured three times to determine the standard deviation (S). With each test series, control measurements were carried out. The obtained results were analyzed statistically. The STATISTICA program was used to analyze the obtained results.

**Figure 3:**
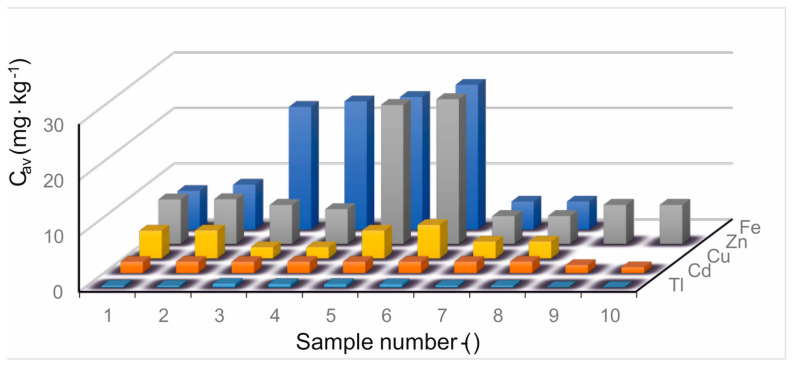
Average concentrations for: thallium, cadmium, zinc, copper, and iron comparison of average concentrations for all samples.

The obtained results of each metal’s content in coal samples are presented in Table 1. It summarizes the results of thallium concentration in the tested coal samples and the average value of concentration (Cav), along with statistical dispersion in the form of absolute standard deviation S and relative deviation (RSD). Control measurements were performed with each series of experiments. Nine independent trials were conducted for the reference material (soil NCS DC 73382) in order to determine the Tl content. The average Tl content was 0.95 ± 0.0967 mg·kg^−1^ (with a minimum of 0.82 and a maximum of 1.10 mg·kg^−1^). The result includes the samples sourced from Poland and from beyond the eastern Polish border. In order to illustrate the results more clearly, the concentrations are compared in Figure 3. The statistical dispersion is small, less than 1%.

The study confirmed the presence of thallium in the tested coal samples; in substance, the coal samples from outside the European Union contained four times more thallium than the samples of Polish coal. This can cause a serious problem, as thallium salts are now considered to be one of the most toxic compounds (1000 times more toxic to the body than cadmium) [4]. Estimating a safety rating is a complex problem when it comes to the mobility of the waste. To prevent thallium poisoning, its content must not exceed the environmentally safe limits, which are 2 µg·L^−1^ for drinking water, 0.008–1.0 mg·kg^−1^ for terrestrial plants, and 0.03–0.3 mg·kg^−1^ for food plants.

In Table 1, the results of cadmium (Cd) samples are also discussed. The value of statistical dispersion is higher than for thallium and reaches 6.5% for the samples of coal sourced in Poland. The tested coal samples from outside the EU are characterized by higher average cadmium concentrations. Moreover, the results for zinc are shown (Table 1). Based on the analysis of the results, it can be concluded that average concentrations are not high in most of the samples. Except for two samples (5 and 6), the statistical dispersion is greater than that of thallium and in one case reaches 3%, with a small average concentration of zinc in the sample (4). The tested coal samples from outside the EU, in most cases, exhibited similar concentrations of zinc to the samples of Polish coal. For the last two elements, copper and iron, it was concluded that in most of the samples they had higher concentrations than the previously discussed elements. The maximum concentration value for copper is 5 ÷ 6 mg per kilogram of coal, and for iron above 25 mg·kg^−1^. In both cases, four out of six tested coal samples from outside the EU significantly exceeded the results obtained for the samples of Polish coal, and the other two showed similar results. Figure 3 shows the results of measurements of heavy metal concentrations in the samples of hard coal sourced in Poland (9 and 10) and from outside the EU (1–8). The results show that the averaging of concentrations of all elements indicates greater pollution levels of coals imported to the EU from outside the eastern border of Poland, which is important because all these elements are emitted in a gaseous or solid form to the environment. In the era of limiting the use of coal, it is essential to follow multi-criteria assessments, considering costs, pollutant concentrations, availability, and other equally key factors.

Mineralization, carried out with the use of concentrated HF and HNO_3_ with H_2_O_2_ addition, turned out to be effective. The tested hard coal was completely mineralized. Mineralization with concentrated H_2_SO_4_ with H_2_O_2_ did not lead to complete coal digestion. The metal with the highest concentration in the tested hard coal samples sourced in deposits from outside the EU area was iron. Zinc was the most abundant in the coal samples of Polish origin. The metal with the lowest concentration was thallium both in foreign and in Polish coal samples. This may be due to the heterogeneous structure of hard coal and its specific matrix. Differences in metal content (especially for samples 6 and 7—Figure 3) may be the result of the origin of the coal and its quality parameters, as well as the heterogeneous structure of hard coal and the specific matrix. The environmental chemistry during the sedimentation of plant and terrigenous material (syngenetic mineralization) has a very important influence on the concentration of metals in coals. It is assumed that the diagenesis and metamorphism of organic matter influence not only the increase in the degree of coalification, but also the process of removal and redistribution of major and trace elements in the coal seam. No correlation was found between the content of thallium and other heavy metals. The determined concentrations in all hard coal samples presented in the article have a negative impact on the environment. Coal exploitation has a significant impact on land, water reservoirs, and infrastructure. The most important effects include pumping out water and the drainage of nearby water reservoirs, the degradation of plant areas, and landslides. For comparison, Table 2 presents the results of analyses regarding the heavy metal content in hard coal samples around the world.

Hard coal is still the main energy raw material in many countries. Its share is as much as 41% in the global use of solid fuels used for energy production. Poland holds one of the leading positions in the production of electricity using hard coal combustion. Due to the large emissions of gases polluting the atmosphere, a reduction in gross generated power by 14,925 MW and an increase in the production of electricity from unconventional sources is planned by 2030. Ecological sensitivity is also becoming increasingly important for current and future generations, manifested by the desire to limit the environmental impact of economic processes, including the production and use of energy. A state of energy sustainability includes the optimal development of stable, affordable, and ecologically rational energy systems. In the process of planning the future fuel structure, there is an attempt to design the development of the country’s energy system in such a way that energy is available in a continuous and stable manner, and so that energy systems are inexpensive and cause the least harm to the environment as possible [17].

## 4. Conclusions

The heavy metal content of coal varies by coal seam and geographic region. Analyses have shown that heavy metals occur in all tested coals from central Europe and western Asia. However, there are significant differences in their content, which is best illustrated in Figure 3, which compares the results for all samples. The comparative analysis shows that thallium is the least common among the heavy metals determined. Moreover, in some cases, as in the case of iron and copper, its presence in coals from Central Europe is negligibly small. Studies have shown that low iron and copper content is accompanied by high zinc content. This means that all coals from the studied regions contain heavy metals, only in different amounts. This fact should be examined in two ways. Firstly, the high metal content in coal may in the future be seen as its source. In times of decreasing resources of precious elements, coal may also become a profitable raw material for their extraction. On the other hand, heavy metals contained in hard coal burned industrially pollute the atmosphere. Research has shown that there are various toxic elements within coals that are released and enter the soil and water through the activities of the coal industry, including hard-rock mining and storage on a massive scale, wastewater discharge, and emissions from coal-fired power plants, which, as a result, present a challenge to health and safety. Compounds based on heavy metals are not biodegradable and have a high tendency to accumulate in the environment. Even their gradual release into the environment associated with reduced emissions can lead to serious environmental pollution, and their prolonged presence in land, air, and water systems can significantly increase the risk of exposure. All methods of converting primary energy, including coal, into more refined forms, especially electricity, have their positive and negative sides, in terms of impact on both human safety and health. Improving the quality of coal is the first in a number of possible solutions, and at the same time one of the most important effective processes. Purifying coal through simple processing and enrichment processes reduces SO_2_ emissions, reduces the amount of waste produced by the power plant, and improves the thermal efficiency of the process (thus reducing CO_2_ emissions). Coal purification is standard in many countries. The use of low ash coals also has a significant effect, with environmental benefit. The latest coal processing technologies can produce coal fuel with an ash content lower than 0.25% and very low sulfur content. This will enable the combustion of coal dust with high efficiency, resulting in ultra-low greenhouse gas emissions and other ecological, operational, and economic benefits.

## Figures and Tables

**Table 1:** Results of chemical analyses of metal concentrations in coal samples, where C = m/(1000·M)—metal concentration in sample, Cav—average metal concentration in samples, S—standard deviation, RSD—relative standard deviation.

Sample Number (-)	1	2	3	4	5	6	7	8	9	10
**Tl**										
**C_av_(mg·kg^−1^)**	0.284	0.268	0.636	0.591	0.558	0.531	0.328	0.297	0.055	0.053
**S (mg·kg^−1^)**	0.282	0.5723	0.006	0.011	0.0377	0.0366	0.0046	0.0044	0.0017	0.0017
**RSD (%)**	9.93	21.37	0.94	1.86	6.7	6.9	1.39	1.460	3.1	3.2
**Cd**										
**C_av_ (mg·kg^−1^)**	1.99	2.002	1.99	2.0	1.99	2.0	1.99	2.0	1.4	1.0
**S (mg·kg^−1^)**	0.0054	0.0	0.0176	0.0	0.0	0.0	0.01	0.0	0.1	0.0
**RSD (%)**	0.27	0.0	0.88	0.0	0.0	0.0	5.02	0.0	7.44	0.0
**Zn**										
**C_av_ (mg·kg^−1^)**	7.98	8.01	6.99	6.23	24.97	26.0	4.99	5.0	6.990	6.990
**S (mg·kg^−1^)**	0.0	0.024	0.01	0.2	0.043	0.017	0.017	0.0	0.020	0.010
**RSD (%)**	0.0	0.29	0.14	3.21	0.17	0.06	0.34	0.0	0.280	0.140
**Cu**										
**C_av_ (mg·kg^−1^)**	4.98	5.0	1.99	2.0	4.99	6.0	2.99	3.02	-	-
**S (mg·kg^−1^)**	0.072	0.01	0.01	0	0.017	0.0	0.01	0.026	-	-
**RSD (%)**	1.4	0.2	0.5	0	0.34	0.0	0.33	0.86	-	-
**Fe**										
**C_av_ (mg·kg^−1^)**	6.9	8.01	22.0	23.0	23.8	26.0	4.98	5.0	-	-
**S (mg·kg^−1^)**	0.8	0.0	1.0	0.86	0.26	1.73	0.01	0.0	-	-
**RSD (%)**	11.59	0.0	4.5	3.7	1.09	6.65	0.2	0.0	-	-

**Table 2:** Concentration of heavy metals in hard coal around the world.

Metal	Place of Sample Collection	Concentration (mg·g^−1^)	Source
Zinc	Kańsk-Achińsk, Russia	9.0	Lebedeva L. et al., 2007 [5]
Zinc	Kuźnieck, Russia	30	Lebedeva L. et al., 2007 [5]
Zinc	Yunnan, China	1.4	Wang X. et al., 2015 [6]
Zinc	Fujian, China	65–135	Ke P.; Wen—Xiong W. 2012 [7]
Zinc	Bohaibai, China	25–32	Ke P.; Wen—Xiong W. 2012 [7]
Zinc	Yulin, China	8.55	Jia J. et al., 2016 [8]
Zinc	Guanbauwushire, Mongolia, China	7–40	Dai S. et al., 2012 [9]
Zinc	Nottinghamshire, UK, coalfield	33	Spears D.A. 2007 et al. [10]
Zinc	Alborzagan, Olang, Iran	62	Taghipour N.; Marshk K. 2015 [11]
Zinc	Razi, Olang, Iran	193	Taghipour N.; Marshk K. 2015 [11]
Zinc	Melech Aram, Olang, Iran	84	Taghipour N.; Marshk K. 2015 [11]
Zinc	Elbistein, Turkey	17.28	Sutan Cicioglu E.; Karayigit A.I. 2015 [12]
Cadmium	Kańsk-Achińsk, Russia	0.3	Lebedeva L.; et al., 2007 [5]
Cadmium	Kuźnieck, Russia	0.5	Lebedeva L.; et al., 2007 [5]
Cadmium	Fujian, China	0.1–1	Pan, K.; Wang, W.X. 2012 [7]
Cadmium	Bohaibai, China	0.07–0.2	Pan, K.; Wang, W.X. 2012 [7]
Cadmium	Guanbauwushire, Mongolia, China	0.085–0.2	Cicioglu, E.S.; Karayigit, A.I. 2015 [12]
Cadmium	Yulin, Chiny	0.05	Jia J.; et al., 2016 [8]
Cadmium	Brazil	0.01–7.99	Duarte A.T.; et al., 2013 [13]
Cadmium	Indie	0.4	Das T.B.; et al., 2013 [14]
Cadmium	Elbistein, Turkey	0.39	Cicioglu, E.S.; Karayigit, A.I. 2015 [12]
Copper	Yunnan, China	70.4	Wang X.; et al., 2015 [6]
Copper	Fujian, China	19–97	Pan, K.; Wang, W.X. 2012 [7]
Copper	Bohaibai, China	25–32	Pan, K.; Wang, W.X. 2012 [7]
Copper	Yulin, China	6.62	Jia J.; Li H.; et al., 2016 [8]
Copper	Yulin, China	6.62	Jia J.; et al., 2016 [8]
Copper	Guanbauwushire, Mongolia, China	10–19	Dai S.; et al., 2012 [9]
Copper	Nottinghamshire, UK, coalfield	47	Spears D.A.; et al., 2007 [10]
Copper	India	14.5	Das T.B.; et al., 2013 [14]
Copper	Elbistein, Turkey	9.97	Cicioglu, E.S.; Karayigit, A.I. 2015 [12]
Thallium	Yunnan, China	0.47	Wang X.; et al., 2015 [6]
Thallium	Guanbauwushire, Mongolia, China	0.06–0.2	Dai S.; et al., 2012 [9]
Thallium	Alborzegan, Olang, Iran	0.8	Taghipour N.; Marshk K. 2015 [11]
Thallium	Razi, Olang, Iran	1	Taghipour N.; Marshk K. 2015 [11]
Thallium	Melech Aram, Olang, Iran	1	Taghipour N.; Marshk K. 2015 [11]
Thallium	Elbistein, Turkey	0.71	Cicioglu, E.S.; Karayigit, A.I. 2015 [12]
Thallium	Germany	2	Berndt H.; et al., 1981 [15]
Thallium	Belchatow, Polend	0.2–5.3	Paulo A.; et al., 2007 [16]

## Data Availability

Data are available in this article.
